# Hydrocele Cured by Friction of the Tunica Vaginalis

**Published:** 1857-06

**Authors:** 


*Hydrocele cured by friction of the Tunica Vaginalis.* Reported by E. S. Sharp, M. D., Resident Physician. Prof. George. C. Blackman, Attending Surgeon.

Sebastian Hook, æt. 59, mechanic, of billious temperament, and regular in his habits; admitted October 17, 1856. About five years before, his scrotum commenced enlarging, which finally increased to a very large size, in the space of two years. After which time the veins of his legs became enlarged, and ulcers formed of considerable dimensions over both tibia. When admitted, his general health was tolerably good. Scrotum much enlarged and fluctuating. Tunica Vaginalis contained probably a pint and a half of fluid. The veins of both legs were enlarged to three times their normal size, with several ulcers over both tibia. Six weeks after his admission his hydrocele was tapped with a trocar, and the above named amount of fluid evacuated. No injection was used, and instead, friction was employed for a few seconds, by Prof. B., causing no inconvenience to the patient at the time. In a few hours the scrotum appeared much swollen, red and somewhat painful. Ordered cold water dressings and opiates at bed time. The inflammatory process gradually subsided, and in a few days the hydrocele was cured.—*Western Lancet.*

